# The digitalisation in chartering business: special reference to the role of e-bill of lading in the bulk and liner markets

**DOI:** 10.1186/s41072-022-00129-2

**Published:** 2022-12-19

**Authors:** Evi Plomaritou, Sotiris Jeropoulos

**Affiliations:** Department of Maritime Transport and Commerce, Frederick University, 18, Mariou Agathagelou Str., Agios Georgios, 3080 Limassol, Cyprus

**Keywords:** Chartering business, Digitalisation, E-bill of lading, Charter party, Chartering negotiation, Pre-fixture, Post-fixture, Fixture

## Abstract

Previous research offering a comprehensive overview of digitalisation in maritime transport is limited. In this paper, the authors use several cases to examine digitalisation’s role in the chartering business. The applications of innovative digitalisation to enhance efficiency in the shipping business are presented analytically. Special emphasis is given to the role of the e-bill of lading in the bulk and liner markets. The advantages, disadvantages and legal barriers of the e-bill of lading are examined thoroughly for both markets (bulk and liner markets). The research follows a qualitative case study approach. It shows that although digital technologies offer important advantages in the chartering business, many legal barriers still need to be overcome. The findings fill the gap in the literature and assist maritime professionals and shipping companies in understanding the necessity of digitalisation in chartering business.

## Introduction

In recent years, firms in almost all industries and sectors have conducted several actions to explore new digital technologies and exploit their benefits. Digitalisation is the transition from an analogue to a digital format; it is a process-driven transition to introduce new digital technology (Matt et al. 2015). Digital transformation is a transformation of key business processes, operations and models to exploit the opportunities created by digital technologies, resulting in improved customer service and value additions to the business. It is an organisational and cultural shift (internally) that allows shipping companies to embrace the positive changes offered by digital solutions to entire processes, competencies and business models.

According to Dehning et al. (2003), digital transformation causes fundamental changes in traditional business practices by implementing and using digital technology. Matt et al. (2015) state that this transformation affects services, processes, organisational structures, management strategies and marketing policies. It causes changes in business processes and enables the creation of new types of organisations, bringing changes in organisational culture, business relationships, value creation, customer satisfaction, and market position (Lucas et al. 2013). Manaadiar (2020) argues that digitalisation refers to technology, whereas digital transformation refers to customers. Therefore, digitalisation deals with information and data and the processes and roles required for business operation, while digital transformation deals with the business and its strategy.

The new technological possibilities to process big data and connect it intelligently using algorithms are resulting in various digital innovations. The most important of these from an economic perspective are (Berenberg 2018, p. 19):

- Digital platforms,
- Virtual and Augmented Reality,
- Artificial Intelligence,
- Internet of Things,
- Blockchain,
- 3D Printing Methods.

Digitalisation portrays two sides like every coin due to its benefits and pitfalls. On the one hand, it refines connectivity possibilities, advances knowledge sharing, improves performance and meliorates corporate efficiency (Eißfeller 2020). Manaadiar (2020) states that creating digital standards related to the exchange of digital information across the value chain improves the potential for digital efficiency. The digital data collection related to safety, emissions, and operational efficiency opens up new possibilities to share data, increasing transparency and creating benchmarks around key performance indicators. Such benchmarks can be used internally by the companies to improve performance and externally by investors, lenders, insurers and customers, to prioritise companies with environmentally friendly or safer business practices, thereby driving productive industry change.

In addition, the spread of data collection technologies combined with the rapid expansion of computing power and artificial intelligence makes it possible to analyse very big data sets, improving operations and forecasting potential risks. Greater amounts of analysable data create better and more comprehensive analytical results. For example, digital data can be used for decision-making, enhanced monitoring, systematic control and quality assurance (Manaadiar 2020). Moreover, another potential benefit of digitalisation and data sharing is to reduce transaction costs by automating the exchange of information between different parts of the value chain. In addition, technologies such as blockchains offer a way of ensuring trust and transparency (Manaadiar 2020; Goldsby and Hanisch 2022). As digitalisation progresses more and more in all sectors of the economy, the maritime sector is also being revolutionised. Because trade, transport and logistics are derived variables, developments and changes outside shipping will most certainly play at least as important a role as the direct changes in processes and business models within the shipping industry.

Considering the tremendous advantages of digitalisation, this research attempts to present the main benefits and barriers (practical, economical and legal) of digitalisation in chartering business. For this purpose, the research follows a qualitative case study approach. It investigates how leading shipping companies in the bulk and liner markets handle the fundamental issue of the new technology. Particular reference is made to the electronic bill of lading role in the bulk and liner markets since the bill of lading plays an extremely important role in sea transportation.

The paper is structured into six sections. “Introduction” section  addresses the introduction; “Shipping market and chartering business” section presents the main elements of chartering business in bulk and liner markets; “Literature review on digitalisation in shipping” section  focuses on digitalisation in the maritime sector and introduces digitalisation in the chartering business (in bulk and liner markets); “Research methodology” section deals with the research methodology;  “Findings” section presents the findings and “Conclusions and recommendations” section displays the conclusions and recommendations.

## Shipping market and chartering business

Companies and organisations engaged in shipping require adequate information and a great deal of flexibility. Apart from the daily fluctuations in freight rate levels and trading conditions, there is a constant development towards new techniques in shipbuilding and propulsion, cargo handling, terminal operations, etc. Furthermore, due to the ever-changing conditions in international commerce, overseas trading patterns change and new cargoes and loading–discharging locations emerge, sometimes quite drastically diminishing the importance of previously busy ports and critical cargo movements. Such changes will occur over a few years and probably several times during a vessels’ commercial lifespan (typically 20–25 years, although sometimes ships may trade up to 30 years or even more). Therefore, irrespective of whether the freight market is booming, flat or depressed, there are also seasonal changes in cargo volumes to be shipped (Stopford 2009).

Within the shipping business practice, ‘*chartering*’ may be simplistically defined as the act or procedure that deals with vessels’ commercial employment, cargoes’ international transport and often their appropriate matching. Chartering or similar sea transport engagements, including booking transportation of cargoes with liner ships, can be based on different methods and principles, as follows (Plomaritou and Papadopoulos 2018, p. 220):

- *From a functional point of view*, the most important distinction of charter types is made among a voyage charter, a time charter, a bareboat charter and some hybrid forms.
- *Chartering vessels in the open market on a charter party basis versus liner services provided on a booking note basis.* In liner shipping, the liner operator generally acts as a common carrier, accepting all general cargo shipped between the ports covered by his service. Terms and conditions of the cargo transport (mostly containers) are agreed on the ‘booking note’, while the contract of carriage is usually the ‘bill of lading’. On the contrary, in non-regular, bulk/tramp shipping, the shipowners continuously seek the best employment for their vessel considering its type, present position, state and expected development of the freight market, etc. Terms and conditions of vessel employment are depicted on a ‘charter party’.
- *Fixed sum versus payment for time spent*. Charter agreements may be divided into two wide groups. On the one hand, project-based agreements, where the owners are paid a fixed sum for doing a specified job, and on the other hand, time-based agreements, where the owners are paid based on the time spent by the charterers for the use of the vessel or part of it. Fixed sum is also called lumpsum. Project-based/fixed sum types of charter are voyage charters and liner services, while the time-based types of charter are time charters and bareboat charters. Contracts of affreightment are the most important of the hybrid charter forms, which cannot easily be sorted under any of the above-mentioned types of charter.
- *Use of the ship from a capacity point of view.* An important basis for distinguishing different types of charter agreements is the use of the ship from a capacity point of view. The charterer may have chartered the whole vessel, that is, all the space of it. Then, the charter party spells out that the charterer shall deliver ‘a full and complete cargo’ to be loaded within the limits of the ship’s capacity. If the owner cannot find a charterer for the whole vessel, he may divide the vessel’s cargo space between several charterers who may each use, for example, certain portions of the vessel or certain cargo holds. This is known as space or slot charter. Space charter is not the same as when several charterers charter the vessel. In the open bulk market, the most common charter types are those concerning the chartering of the whole vessel. On the other hand, in the liner business, the owner (carrier) normally promises to carry a specified cargo (e.g., 10 boxes of machinery, 100 bags of coffee etc.), among many other cargoes carried simultaneously, typically in containers. This is known as the carriage of general cargo.

In such a dynamic commercial environment, shipowners, ship managers, fleet operators, charterers and shippers, shipbrokers, and agents must take advantage of new opportunities to survive and remain competitive. Therefore, digitalisation is an excellent tool for improving companies’ competitive position in the charter market.

### Chartering business in bulk shipping

The chartering business in the bulk market is divided into three phases: the pre-fixture phase of the charter, the fixture phase of the charter and the post-fixture phase of the charter. Chartering is a part of the commercial management of ships, which, however, cannot exist by itself. The operative force in the background is always a sales contract of goods and the need for sea transport. So, first, there is a sale/purchase of merchandise; second, a need for sea transport; and third, a need for chartering/hiring a vessel. Therefore, the pre-fixture of a charter is constituted of three stages. The stage of the sale of goods, the stage of investigation and the stage of negotiation. The fixture includes the stages of the drawing up and the signing of the charter party. The post fixture of a charter includes the stages of carriage of goods by sea. More specifically, the post-fixture of a voyage charter is constituted of the stage of ballast (or preliminary) voyage, the stage of ship’s arrival at the port of loading, the stage of loading operation, the stage of issuance of bill of lading, the stage of laden (or carrying) voyage, the stage of ship’s arrival at the discharging port, the stage of unloading/discharging operation and the stage of delivery of cargo to the cargo owner (Plomaritou and Papadopoulos 2018).

### Chartering business in liner shipping

In the liner market, concluding a transportation agreement involves the pre-booking, booking and post-booking phases and bears some similarities to the process of the bulk market. However, the essential difference between a pre-fixture and a pre-booking phase is that in the latter case, a booking procedure takes place instead of the negotiation procedure, leading to the issue of a quotation code and the booking note and not the signature of the charter party. Besides, this procedure occurs between the liner agent and the shipper or the freight forwarder instead of the shipowner, the charterer and their brokers. Additionally, the fundamental difference between a ‘bulk market’ post-fixture phase and a ‘liner market’ post-booking phase is that the latter is more likely to include logistics integration solutions and door-to-door services offered to the owner of the cargo. Promoting this vertical diversification, i.e., providing more door-to-door services instead of only port-to-port, is part of most lines “current strategy” (Bockmann 2022; Baker 2022; Concepcion 2022).

Digitalisation is not something new for container lines. With the introduction of global systems that each container line introduced in the early nineties, the container business changed dramatically, even if the change was not obvious to the cargo owners. Entities like the Digital Container Shipping Association (DCSA,) TradeLens, the Ship-planning Message Development Group (SMDG) and the TIC 4.0 were, at that time, still in the far future (Lind et al. 2021). One of the most important developments in the liner market is the rise in the digitalisation of freight enquiry, quotation and booking software. Digitalisation of sea freight booking through automation and cloud-based technologies is showing unprecedented growth in the digitalisation of maritime freight market (Hirata et al. 2022).

Advances in digital technology enabled data exchange and communications to progress far beyond sending bills of lading and manifests by courier services or facsimile transmissions from the port of loading to the port of discharge across the globe. By 1995, the loading port agent could enter all data relevant to their exports in the container line’s global system. This information became immediately accessible and useable by the discharging ports. It was no longer necessary to re-enter the same data for the other process steps. The quotation, booking, bill of lading, loading and discharge lists, cargo and freight manifests, export and import invoices and delivery orders have since become part of a single data chain (DCSA 2020).

The digitalisation of freight quotations, bookings, and other similar procedure steps may not have been extensively researched (Balci 2021) but are already widely used. Repetitive entry of the same data is by now completely obviated. Data includes that on the bill of lading and all invoicing amounts, freight-related and other. All this information is available for the accounting needs at every point of the container line network, including headquarters for control and reporting, through the global systems that each container line uses. Bearing in mind the global tendency for container lines to own their agency networks worldwide, the headcount necessary to carry out the necessary steps to serve international trade has significantly reduced. This technology is available to every container line in operation today. Conversations with the interviewees reconfirmed these already well-known liner shipping practices.

### The role of e-bill of lading in the bulk and the liner markets

In bulk shipping, the charter party is the contract of carriage between the shipowner and the charterer. In the hands of the non-chartering shipper, the bill of lading has no contractual capacity at all as shown in *Rodoconachi v Milburn (1886) 18 QBD 67, CA; President of India v Metcalf Shipping [1970] 1 QBD 289* (Voudouris and Plomaritou 2020). Charter party terms determine the rights and liabilities of the contracting parties (Plomaritou 2014); Bills of lading are issued upon shipment of the goods. Therefore, the bill of lading cannot vary or add to the terms of that charter party unless the charter party contains an express provision to that effect. However, the bill of lading contains the carriage terms and conditions if it is endorsed and transferred to a subsequent consignee (Anderson 2018). Once the bill of lading is endorsed (transferred) to a third party (consignee, endorsee, or transferee), it is conclusive evidence of the contract of carriage. Any oral or written agreement which the shipper and the shipowner (carrier) have and which is not expressed in the bill of lading will not affect the third party (Plomaritou and Voudouris 2019). The reason for this is that the third party does not notice any of the terms that the shipper and the carrier may have agreed to orally and that have not been expressed in the contract of carriage.

The liner operator acts as a common carrier in liner shipping, accepting the general cargo shipped between the ports covered by his service. A shipper, who wishes to use only a part of a vessel, contacts the agent of a particular line, who then confirms the booking of cargo space onboard the ship by issuing the so-called ‘booking note’, or ‘shipping note’, or else a ‘fixture note’. Unlike the charter party, in English law, this initial contract is not definitive of the contractual terms. These will be fleshed out by the terms of the carrier’s usual bill of lading (Voudouris and Plomaritou 2020). This may happen expressly, as in *Armour & Co Ltd v. Walford* (1921, 3 KB 473) (www.i-law.com/ilaw/doc/view.htm?id=136901), or impliedly, as in *Pyrene Co Ltd v. Scindia Navigation* (1954, 2 QB 402) (www.i-law.com/ilaw/doc/view.htm?id=145111). So, when the goods have been received for shipment or shipped on board the vessel, a bill of lading will be issued on behalf of the carrier. Consequently, the bill of lading evidences the contract of cargo carriage between the carrier and the shipper (or the endorsee).

Electronic bills of lading have been in use for several years. Bills of lading produced in electronic format are designed to replicate their paper’s purposes and processes (such as endorsements or reservations) equivalent to offering ‘functional equivalence’. If required by parties in the trading chain, electronic bills can be replaced by paper bills of lading at any point. In practical terms, while the electronic bill of lading systems do eliminate the problem of cargoes arriving at discharge ports before hard copies bills of lading, their use significantly reduces the number, associated risks and costs of all methods of delivering the cargo without an original bill of lading (Plomaritou and Nikolaides 2016). Among the various benefits of electronic bills of lading are an increase in speed and a reduction in the cost of transactions, together with the elimination of the problem of the late arrival of documents (Wilson 2010).

In 2010, the International Group of P&I Clubs (IG), comprising thirteen P&I Clubs, approved the Bolero and the essDOCS systems before joining the e-title system in 2015. The IG started covering liabilities arising in respect of the carriage of cargo under electronic paperless trading systems from 20 February 2010 onwards and approved four electronic paperless systems as below:

- Bolero by Bolero International Ltd—Rulebook/Operating Procedures September 1999;
- CargoDocs by Electronic Shipping Solutions;
- e-titleTM by E-Title Authority Pte Ltd;
- edoxOnline by Global Share S.A.

Consequently, if any liabilities occur on goods shipped under other electronic paperless trading systems (not approved by the Group), the members will not be covered for this.

BIMCO (2014) has for many years fully supported the concept of electronic bills of lading to provide a more efficient, systematic, organised and secure method of dealing with bills of lading and other transport documents like delivery orders (UNCTAD 2003). However, the increasing use of electronic documentation, particularly in the bulk market segment where many major charterers actively promote it, has resulted in a growing user requirement (from shipowners and charterers) for BIMCO to develop a specialist provision. In response to this demand, BIMCO brought together a group of charterers and shipowners to develop a new clause for charter parties that specifically addresses electronic bills of lading (paperless trading) systems. This electronic bill of lading clause needs to be incorporated into charter parties when the contracting parties (shipowner and charterer) know that an electronic bill of lading will be issued. More specifically, the BIMCO Electronic Bill of Lading Clause is the following (BIMCO 2014):

“(a) At the Charterers’ option, bills of lading, waybills and delivery orders referred to in this Charter Party shall be issued, signed and transmitted in electronic form with the same effect as their paper equivalent.

(b) For the purpose of Sub-clause (a) the Owners shall subscribe to and use Electronic (Paperless)Trading Systems as directed by the Charterers, provided such systems are approved by the International Group of P&I Clubs. Any fees incurred in subscribing to or for using such systems shall be for the Charterers’ account.

©The Charterers agree to hold the Owners harmless in respect of any additional liability arising from the use of the systems referred to in Sub-clause (b), to the extent that such liability does not arise from Owners’ negligence.

Under sub-clause (a) of the BIMCO clause, owners and charterers agree that the eBL issued will have the same effect as a paper BL.”

The advent of blockchain could prove a renaissance for the e-bill of lading. The Bolero electronic bill of lading was successfully used in a blockchain transaction, and in the near future, electronic bills of lading are expected to substitute the traditional bills of lading. Blockchain is expected to play a significant role in the digitalisation of the bill of lading in bulk and liner markets. Blockchain may be considered the next electronic data interchange level (EDI). It is a technology where the data and systems are not centralised on a single server. Instead, information is shared among multiple computers that compile a secure network requiring each to be individually hacked to access the whole system (Schleyerbach and Mulder 2021).

Recent success stories of trialling blockchain technology in bills of lading transactions include the Smart B/L from Cargo X, Wave and the Maersk-IBM partnership TradeLens (Forster and Davies 2019). The CargoX trial involved a shipment of garments from China to Slovenia. They report that a bill of lading was successfully processed in minutes at the cost of US$15 using a public blockchain network. The TradeLens system does not just limit itself to the bill of lading but the whole process of transactions and document control in the supply chain (Wass 2017). When studying the need to improve the process, Maersk claimed that a single shipment of avocados from Mombasa to Rotterdam involves 30 parties of over 100 people and 200 information exchanges. Their trials showed a time reduction of 40 per cent and a reduction of costs by thousands of dollars (Maersk 2021).

A blockchain-based bill of lading has advantages over previous e-bills (Cadwalader, Wickersham & Taft 2017; Shope 2021). The decentralisation provided by blockchain technology significantly diminishes human mistakes that the registry administrator might otherwise create (Prieto et al. 2020; ReedSmith 2019; Ζiakas 2018). The system is less vulnerable to hacking attacks, decreasing the risk of fraudulent transactions. Therefore, blockchain technology improves trust in the accuracy and integrity of transactions through automated payment processes. Moreover, the blockchain system diminishes delays in international trade through standardised practices and improves the process of cargo claims, which means lower expenses (Yang 2019).

However, launching a blockchain-based bill of lading presents several major obstacles. Thus, technical matters should be arranged, and legal problems should be overcome. Blockchain technology is a closed, member-only system based on a registry administered by a trusted intermediary. All parties must be registered members to make transactions on a closed platform (Takahashi 2016). When a non-registered member is involved, an electronic bill of lading must be replaced by a paper-based bill of lading. This membership requirement is considered a major obstacle to using electronic bills of lading. In addition, since blockchain technology is decentralised, and has no single controlling entity, liability would not be clear if the system was to fail.

A blockchain-based bill of lading, like any other electronic bill of lading, will not succeed without the approval and cooperation of the relevant legal structures. Currently, English law does not recognise an electronic bill of lading as a negotiable document of title. The electronic bills of lading have not been regulated under the enforced International Sea Conventions, namely the Hague-Visby Rules and the Hamburg Rules. The legal efficacies of digital bills of lading are not fully tested under the law of contract. Even though the present study examines the legal regime of the e-bill of lading under the International Sea Conventions, at this point, a special reference should be made to the jurisdiction of Malaysia and other places which recognise an electronic bill of lading as a negotiable document of title. Although a positive step, the acceptance of the use of e-bill of lading in specific parts of the world is insufficient in an industry as international as shipping.

Consequently, the holders of electronic bills of lading issued under COGSA 1992 will not be able to pursue claims against the issuing carrier unless there is an express contractual agreement covering this aspect (Plomaritou and Voudouris 2019). So, the legal framework protecting carriers who carry cargo under an original bill of lading is not suitable for electronic bills of lading. Furthermore, the lack of adequate support from the legal infrastructure causes many problems with electronic bill of lading schemes, resulting in banks’ refusal to consider electronic bills of lading as adequate collateral. A possible solution to these problems may be found in the Rotterdam Rules, where electronic bills of lading are called ‘*negotiable electronic transport records*’ (Tseng 2017). The Rotterdam Rules 2009 is the most recent attempt of the United Nations to bring legal harmonisation and legal certainty to the international carriage of goods by sea. However, since the Rules have not yet received a sufficient number of ratifications (due to various criticisms), the privately devised rules introduced by the BIMCO and the insurance cover provided by the International Group of P&I Clubs, the conditions in the shipping market vary significantly.

## Literature review on digitalisation in shipping

Digitalisation in the maritime sector uses digital technologies—like blockchain, artificial intelligence, machine learning, automation, etc.—to convert the shipping business into a digital business. The digitalisation of the shipping industry is about separating access to data from ownership of the vessels. On the other hand, digitisation is the process of converting information from a physical format into a digital one. For example, scanning a bill of lading or converting a charter party to a PDF document. The data is not changed—it is encoded in a digital format. Digitization can reap efficiency benefits when the digitized data is used to automate processes and enable better accessibility—but digitization does not seek to optimize the processes or data. Considerable work has been made regarding the digital transformation of the shipping industry, but no extensive research has been carried out regarding the digitalisation of chartering business.

Following Sanchez-Gonzalez et al. (2019), digitalisation currently applies to eight digital domains: “*autonomous vessels, robotics, artificial intelligence, big data, virtual reality, internet of things; the cloud and edge computing, digital security, 3D printing and additive engineering*”*.* Tijan et al. (2021) consider that the shipping industry is moving towards digitalisation and digital transformation at different speeds in different domains and market segments. It is noted that the shipping sector has been slower to adapt to new technology and digitalisation than other industries (ABB 2019). Zaman et al. (2017) argue that the slow adoption of digital technologies is due to many reasons like the longer lifecycle of assets, the high costs of new digital solutions and the strict maritime regulations that prohibit incentives to invest. Vessels are long-lasting assets that have been in operation for up to 30 years with traditionally slow lifecycle investments (ABB 2019). In contrast, digitalisation is a fast-moving, disruptive trend with short innovation cycles.

Next, shipping companies are invited to incorporate something rapidly evolving into a more traditional and complex industry. Retrofitting a long-lasting asset repeatedly with constantly updated technology can certainly be costly and disruptive. Furthermore, shipping is a volatile, risky, unpredictable, cyclical industry for reasons like energy price fluctuations, freight fluctuations and regulations expected to become a lot stricter. In addition, another characteristic of the shipping industry is the highly competitive attitude, which makes the collaboration of companies with competitors difficult. Moreover, shipping industry leaders are conservative, still treading cautiously when it comes to investment in digitalisation (Clayton 2021). As a result, many shipping companies, chartering companies, shipbrokers, freight forwarders, ports, financial institutions and other private entities are working on various digitalisation of their business processes.

Therefore, the relatively slow adoption of digital technologies in the maritime sector cannot be attributed to a lack of benefits. On the contrary, several problems could be solved, and obstacles could be overcome by using digital technologies, thereby contributing towards making the maritime industry safer, cleaner, and more efficient. Digitalisation’s potential in terms of preserving the lifetime of assets, optimising the performance of assets, and increasing the safety, reliability and efficiency of operations means that (if executed correctly) it can enable shipping companies to flourish in this new business environment (ABB 2019). Furthermore, exploiting the power of technology to transform data into useful information can help the shipping company maximise its assets’ value. According to DNV (2021), another benefit of digital technologies to the maritime industry is the boost of decarbonisation for achieving minimum emissions from international shipping.

On the other hand, digitalisation involves risks and pitfalls for the shipping industry. More specifically, it raises new social and environmental problems that require solutions. These include data protection matters and issues involving the use of artificial intelligence. Eißfeller (2020) states that to avoid this pitfall, the engagement of shipping companies in corporate digital responsibility is necessary. The responsible and transparent collection and processing of data is an important aspect of digital ethics. Corporate digital responsibility goes beyond legal requirements since it requires the shipping company’s active involvement in forming a digital world based on ethical principles (Eißfeller 2020). It should be noted that most maritime leaders and decision-makers are not experts on digitalisation, so even though they desire to make the digitalisation, they will need to bring in new skills to do so effectively.

Also, increased digitalisation in the maritime industry means the industry will have to manage increased cyber risks. So, while digitalisation can be used to improve safety, it also creates susceptibility. Therefore, a careful evaluation of cyber risks and their prevention is necessary. Soegaard and Wheeler (2019) argue that despite the challenges that shipping companies must address to obtain the benefits of digitalisation fully, the industry is ready to overcome the obstacles and take the next steps towards digitalisation and cross-industry collaboration. This trend is also apparent in the content of Rotterdam Rules where the electronic bills of lading are called “*negotiable electronic transport records*”.

Conclusively, it could be said that the main motives of digitalisation in shipping are the reduction of costs, making it easier to comply with the multiple regulations in the maritime industry, the proper handling of a large quantity of data and the increase of business effectiveness. Conversely, the main barriers to digitalisation are the high implementation costs, the low quality of offshore internet connections, the lack of investment initiatives, the low level of modern digital technology diffusion through the supply chain and the risk prevention (Tijana et al. 2021).

Literature specific to digitalisation in chartering business is very scarce. Parallels can also be discerned with other relevant industries, notably banking. Liu et al. (2011) for example report resistance and unwillingness from the user’s side to keep up with digital advances; this tallies with findings presented below. Furthermore, according to recent research, the e-bill of lading is currently not acceptable as part of the Letters of Credit procedures in many parts of the world, notably Europe (Michael 2022). This paper tries to fill the gap in the literature dealing with digitalisation in chartering business (in bulk and liner markets).

The present study investigates if digital technologies may provide opportunities and benefits at every stage (pre-fixture, fixture and post-fixture) of chartering business in bulk shipping as well as at every stage (pre-booking, booking and post-booking) of chartering business in liner shipping. The research has shown that chartering business is becoming more efficient thanks to digital technologies (such as the *“Signal Ocean Platform”, *www.thesignalgroup.com) which leverage big data, private shipping data and artificial intelligence (AI) to uncover industry trends and provide a unique market view to shipowners, charterers and ship brokers. As a result, the market players in chartering business are able to analyse critical shipping data and make optimal chartering decisions for their business. Shipowners, charterers, liner operators, shippers, shipbrokers, and forwarding agents may have access to shipping market information, check the commercial operation of a vessel, assess the vessels’ availability, view the vessels’ activities, inspect loading and discharging operations, get the right vessel at the right freight rate, collect information about ports and routes, check the network of lines, estimate profitability per voyage (TCE), maintain up-to-date databases of fixtures, facilitate relationships with their clients etc.

## Research methodology

In order to fill the gap in the literature regarding digitalisation in chartering business (in bulk and liner markets) and get a clear picture of its advantages and disadvantages, qualitative research was conducted in March 2021 involving fifteen shipping companies. Specifically, the survey included seven cases of shipping companies operating in the bulk market and eight in the liner market. In addition, intensive individual interviews took place with a small number of respondents to explore their perspectives on the particular subject of digitalisation. However, the small number of participants in this research does not cause problems with findings’ validity and reliability since the research is a multi-case study which makes a benchmarking in shipping business.

More specifically, the selected enterprises are leading shipping companies globally that apply segmental concentration and offer sea transport services in bulk and liner markets. The companies are well known for business and technological excellence and are listed at the ‘Top 100 shipowners of Lloyds List’. Since they are considered leaders in their operations, they may be used as benchmarks against other shipping companies or other sectors of the shipping business. The criterion of the companies’ selection was that the selected companies constitute standards of organization/ management in the international shipping industry. Benchmarking aims to identify areas, systems, or processes for improvement in the chartering business. This multi-case study of shipping enterprises with proven digitalisation activities was conducted to thoroughly examine the benefits and problems of digitalisation in the shipping business (in bulk and liner markets). Based on research ethics and for confidentiality reasons, the anonymity of the interviewees was preserved.

The in-depth interviews were done with key interviewees from the chartering departments of the bulk shipping companies and the operational departments of the liner shipping companies. The interviews were based on open-ended questions (free-form survey questions) that allowed respondents to answer in open-text format. In this way, the responses were not limited to a set of options, providing valuable information about the subject at hand. The responses to the questions offered detailed and descriptive information on digitalisation in chartering business. The questions set to the shipping companies of the bulk market were similar to those set to the shipping companies of the liner market, except for some minor differences due to the different character of the businesses in those markets. For example, the questions made to the bulk shipping companies were oriented to the stages of pre-fixture, fixture and post-fixture of chartering business, while the questions made to the liner shipping companies were oriented to the stages of pre-booking, booking and post-booking stages. Since there is no rule regarding a compulsory number of questions that must be set for the interviewees, the researchers decided that a minimum number of 10 questions should be set for the interviews.

The interview was constituted of two parts. The first part concerned digitalisation technologies in development or use. This part included eight questions, which means one question for every stage of pre-fixture (or pre-booking), fixture (or booking) and post-fixture (or post-booking) of a charter. The second part concerned the benefits and pitfalls of the technologies mentioned above. This part included two questions, one for the benefits and one for the pitfalls of digitalisation. More analytically, the interviewees were asked to present how their companies applied some digitalisation technologies. Special also reference was made to the electronic bill of lading. In addition, the interviewees were asked to explain and comment on any possible benefits and/or pitfalls created by implementing digitalisation in the chartering department. The selected interviewees were expected to provide reliable information regarding shipping digitalisation, as they are experts and well-known in chartering business and information technology. All interviews took place face-to-face, at the companies’ premises. Furthermore, secondary data was collected and analysed, such as companies’ brochures and additional material provided by interviewees.

## Findings

### Digitalisation in the chartering business of the bulk market

The following sections present the research findings regarding the implementation of digitalisation in the bulk market chartering business. More specifically, the findings are presented for every stage of a charter’s pre-fixture, fixture, and post-fixture. Furthermore, reference also is made to the electronic bill of lading.

#### Digitalisation in pre-fixture and fixture stages

At the question of which digitalisation technologies are in development for the pre-fixture and fixture stages, the interviewees answered that over the years, there were featured various web-based platforms offering e-chartering services (e.g., Signal Ocean Platform, Soft Ship, Gat Ship, Safe Sea Net, National Maritime Single Window /NMSW, AXS Marine, ShipNext, CPVault, Magellan Chartering Solutions, ShipFix, Veson Nautical, EdoxOnline etc.). These e-chartering platforms offer a range of applications such as maritime news, market forecast, fixture reports, ports’ information, voyage estimations, laytime calculations and other pre- and post-fixture applications. In this way, a software platform that has been developed side-by-side with shipbrokers, charterers and shipowners leverages artificial intelligence to turn complex data into meaningful market insights for brokers and other market participants (www.thesignalgroup.com/newsroom/digital-transformation-shipping-data). Especially a shipbroker—who acts as a middleman between the shipowner and the charterer during the chartering negotiation—to be successful, needs to quickly and efficiently consolidate and analyse critical shipping data provided by the digital platforms (Signal Group 2020a, b).

Regarding the benefits offered by digitalisation at the stages of pre-fixture and fixture of a charter, the interviewees mentioned that many of these platforms meet the market’s expectations. These platforms aim to reduce the number of emails sent and received among shipping market practitioners, saving up time and facilitating a smooth negotiation process. Instead of sending out emails, users can set up various groups and give permission to see open cargoes and positions. In this way, the other users do not need to read many emails, but they have the information available and can find what they need. Chartering negotiations may be saved in a secure file with an automated recapitulation of all agreed terms. Electronic negotiations conclude fixtures.

However, despite the benefits of chartering platforms, the interviewees suggested that a global e-chartering platform must be created and managed by a credible independent organisation to ensure that no company has an advantage over other competitors. After completing each voyage, the global platform should allow charterers and owners to give a rating, evaluating the counterpart’s performance. After many such fixtures, the average rating would become quite representative. An online global environment may help the shipping industry become more transparent and competitive. In this way, shipping will also become less personal and more standardised.

Furthermore, some interviewees use online solutions that digitally capture charter party documentation and information for contract management. These online tools initially digitise paper-based charter documentation and then store it in an online repository. The data is hosted at an International Organization for Standardization (ISO) certified and Statement on Standards for Attestation Engagements (SSAE) audited facility within the European Union and is accessible from anywhere. A dashboard provides an overview of charter documentation, and users are provided with version control and history, as well as drop-down menus and email notifications.

#### Digitalisation in post-fixture stages

After the charter fixture, the carriage of goods by sea stages follows. Again, digitalisation offers many benefits at the stages of post-fixture as well.

At the questions of which digitalisation technologies are in use at the post-fixture stage and the offered benefits, the interviewees mentioned that they use integrated software solutions, ranging from vessel requisitions to company financial statements and prompt, reliable and cost-effective ship-shore communication systems. Five companies overcome technical problems in ship-to-shore connectivity by deploying digital systems, like Inmarsat Fleet Xpress, throughout their fleets to provide fast and reliable connectivity between ships and management offices. These systems allow them to use office applications onboard the vessels.

Five interviewees stated that they have digital systems allowing detailed performance monitoring. More specifically, the companies use high-technology ship management applications that automatically input data from ship to shore, logistics monitoring, continuous training, safety control, the ships’ maintenance and the fleet’s performance and management.

Three interviewees declared they implemented enterprise resource planning (ERP) and fast Ka-band satellite communications. A fully integrated ERP at offices and vessels covers tasks related to trading, chartering, voyage planning, voyage estimation, projection, operation, vessel performance, port calls, electronic invoicing, accounting, insurance, payments and banking, crew management, technical management, planned maintenance, risk management, audits and vettings.

The above systems collect real-time data from sensors onboard the ships or from the main engine and other machinery. Then, these data are used to produce real-time reports or alerts. Furthermore, these data are used to predict serious failures. In addition, an intelligence platform is used for the information in the ERP system to be further analysed, and more sophisticated reports to be used by management as decision support tools. Other benefits these systems offer include profitable bunker management (accurate calculation of bulkers and consequent reduction of bunker consumption), engine performance optimisation, profitable voyage planning (based on actual weather conditions), etc. Furthermore, timely damage prevention will save the company from drydocking expenses, towing operations, off-hire claims and other unpredicted expenses.

All interviewees admitted that any investment in digitalisation and associated technologies deliver competitive advantages and cost benefits for shipowners and managers. They consider that digital technologies like blockchain, the internet of things (IoT), artificial intelligence, machine learning etc., in combination with modern connectivity methods, may provide prevention of system failures, reduction of maintenance costs, a decrease of off-hire claims, the accuracy of the information, compliance with maritime regulations, control of security and availability of data. Further adoption of IoT, artificial intelligence (AI), and machine learning in the shipping industry could facilitate these benefits for all stakeholders. In other words, digitalisation may bring a real revolution to shipping in the near future.

#### Digitalisation and bills of lading

When asked about the use of e-bills of lading, the interviewees answered negatively to the question related to the use of electronic bills of lading. This is due to the cyber risk, the ability of electronic bill of lading to function as a document of title, the transferability problems, the ambiguity of the legal status of e-bills of lading in jurisdictions, the high investment cost, the long hours of training etc.

### Digitalisation in the chartering business of the liner market

As mentioned above, in preparation for this study interviews were taken from eight leading container lines, jointly representing more than 67 percent of the international market share.

#### Digitalisation in the pre-fixture (pre-booking) and fixture (booking) stages

In the container lines market, these stages involve a) either the acceptance by the cargo owner of the container lines tariff or the negotiation of an acceptable freight rate, giving rise to a unique quotation code, and b) a booking note and or shipping instructions sent by the cargo owner (or their forwarder) to the container line using the said code. Technology has been in use for at least 2 decades, and this procedure can be done online using the container lines’ systems. This process utilises data entered by the cargo owner ab initio, and obviates double data entry by any party at any stage of the very long documentation chain until cargo is delivered to the final receiver. Interviewees unsurprisingly reported some shippers’ resistance to taking part in this process, insisting on sending bookings/shipping instructions by fax or email, thus obliging the loading agent to enter the data in the container line’s system.

A defence in attitude among container lines is notable even at this early stage. Some container lines simply no longer acceptable to cooperate with shippers unless the shipper is willing and able to liaise with the container line solely through the container line’s digital channel. As a result, the container line will either refuse their business altogether or encourage this shipper to use the container line’s service via a forwarder who fully uses the container lines’ systems. It is even possible that this service, the ‘digital’ aspect of liner documentation, is offered as part of added logistics services by the forwarder to the cargo owner.

#### Digitalisation in post-fixture (post-booking) stages

In the liner market, once the freight rate has been agreed upon, a booking made, and shipping instructions sent by the shipper, the bill of lading is issued. Then, the information entered by the shipper (or forwarder) is checked and verified by the loading agent, and the Bill of Lading is ready to be issued.

Interviewees reported that the facility allowing selected and pre-approved shippers to print the original bills of lading at their premises did not prove widely successful. Interested exporters were given access to parts of the Maersk system, which enabled them to print the original bill of lading at the exporters’ premises. This removed the necessity of sending hard copies of the most important document in the procedure from the Line’s premises to the shipper. However, this step was met with insufficient acceptance and was mostly abandoned, before the recent cyber-attacks. Most exporters preferred to deal with the bills of lading in the traditional, analogue, hard copy way. Notably, most payments continued to take place physically by the customs broker issuing a cheque at the container line’s agency premises.

Avoiding printing the bills of lading at their premises was not the only way that the customers of container lines, exporters, and importers showed their reluctance to adopt the digital channels of the necessary process. This resistance to digitalisation appears to be more pronounced in less developed, smaller markets and by smaller clients. All interviewees reported that customers with smaller volumes seem to be finding it easier and prefer to liaise with freight forwarders in a more analogue mode rather than deal with the digital profile of container lines. This is covertly encouraged by some container lines. The forwarder then undertakes the electronic exchange of data with the different systems of each container line, thus utilising and offering to the smaller cargo owners’ economies of scope. During the interviews that took place in preparation for this research, every major container line reported that the main reason digitalisation and the paperless procedures have not progressed faster is the resistance on the part of the cargo owners, either because of trust and confidence issues on the paperless processes or because of insufficient information technology (IT) skills on their part. This is also supported by literature (Liu et al. 2011, Schleyerbach and Mulder 2021) reporting unwillingness from the user’s side to keep up with the digital advances.

The correct handling of the bill of lading is key to any successful migration to a completely paperless procedure for containerised exports and imports. Container lines and their customers have found ways to implement paperless procedures without waiting for the resolution of all legal and other impediments (Richardson et al. 2021) to the electronic bill of lading roll-out. These ways are more easily applicable when the specific trade does not involve a letter of credit, and a bank is not part of the process (Marxen 2020; Wass 2019).

Every interviewee mentioned the so-called ‘telex release’ method as a means very frequently used today to release cargo to consignees without presenting an original bill of lading. The term refers to the outdated use of the telex machine as a means of communication, now replaced with other electronic means of communication like email. For most carriages, bills of lading are issued without involving a bank, without a bank being named as shipper or consignee, and without necessitating endorsement for the transfer of cargo ownership from the mentioned consignee to another. In most carriages, the bill of lading and the consignment are not held as collateral nor become part of a Letter of Credit procedure. In these cases, the ‘telex release’ is a shortcut to the procedure, which is very frequently used by all the container lines in this study.

As also outlined in the literature (MOL, Mitsui OSK Lines 2021), the process can be briefly described as follows: the exporter provides the information necessary to the loading agent to issue the bill of lading. This information is entered into the container line system, and the bill of lading is either not printed at all or printed but not handed over to the exporter, it is kept at the issuing agent’s premises. Either way, the physical cargo transport proceeds normally. When the exporter receives payment for the transported goods and is ready to release the goods to the receiver, after settling any outstanding amounts between them and the agent, instructs the latter to release the cargo to the receiver. The agent can then proceed in different ways: he can change the character of the bill of lading in the container line’s systems to show that the bill of lading is not negotiable but a sea waybill instead. The cargo receiver is not obliged to surrender an original document to the delivery agent. Alternatively, the load port agent can instruct the delivery agent to release the cargo to the receiver, indicating that the bill of lading is ‘accomplished’ and in their possession. In support of such instructions, container lines usually ask that these be accompanied by a scan of the original bill of lading and the shipper’s written instructions.

These are procedural shortcuts that offer solutions in cases of payment or other delays in the documentation chain and can help the timely release of cargo at the destination, even in the frequent cases of very short transit time. The exporter needs to be comfortable using this solution in terms of trust and confidence in this paperless process and the chosen container line. This method does not require a high level of IT competence from the side of the exporter; it is, therefore, more widely available than other digital solutions and applications. It is also more widely accepted by shippers who remain hesitant to go further with digitalisation.

#### Digitalisation in the wider liner business

Exchanging information is not limited to the container line internal network. All interviewees took time to talk about how electronic ‘bridges’ are put in place today by using EDI files to facilitate communication with other entities external to the container operator. The exchange of these files connects the container lines’ systems with those of the terminals, port and customs authorities. The output of one system, if in the appropriate format, the output of one system can be the input in another. Delivery orders for empty and full containers are communicated electronically in real-time by the container line to all appropriate parties. The container line equally well receives information entered by terminals and, increasingly, customs authorities. Increasing digitalisation of entities such as customs authorities and container terminals facilitates and accelerates the container lines’ digitalisation process. The benefits in efficiency and speed are here without any downside, every stakeholder reaping only gains.

In greater detail, discharging and loading plans are exchanged between the container line and the terminal to plan port operations. This is done electronically today, when, until very recently, manually prepared plans, highlighted in various colours and manually crossed out with pen and pencil, were given in hard copies to every party involved. Dangerous or awkward cargo information is sent by the container line to the terminal well in advance electronically. The terminal plans its position according to the nature of the cargo and the cargo already stored in the terminal. Thus, control by the competent authority of dangerous cargo regulations adherence is made simple because of the electronic means available today. However, there is no uniformity to these electronic ‘bridges’ used so widely today among terminals, container line, customs authorities, and agencies. Purpose-built solutions are reportedly in use very frequently worldwide, although significant efforts are being made for uniformity and standardisation (https://dcsa.org, https://smdg.org/).

All interviewees reported that the pandemic provided an excellent opportunity to persuade cargo owners to use electronic means to cooperate with the container lines when the latter had to prohibit physical visits to their premises. Payments were only allowed by bank transfer; delivery orders were given electronically; containers were released using the EDI links discussed above. This demonstrated to the reluctant cargo owners that digitalisation works and is trustworthy. Visible improvements in efficiency and speed also became evident to all stakeholders. However, it remains to be seen if the digital means to manage transport activity continue to be preferred after the pandemic. Container lines may continue to insist on these means in the future to maintain improved efficiency and lower costs supported by the paperless processes.

Attitudes differ among container lines about digitalisation. Some container lines voiced concern claiming that the electronic means of conducting business obviate personal contact, weaken customer relations and reduce customer loyalty. Although it is commonly accepted that digitalisation is here to stay and will eventually be the only way to accomplish all container transport steps, container lines disagree on the degree of persuasion or coercion to be applied to the customer. In bigger, more advanced markets, a gap is becoming apparent where freight forwarders are allowed, even encouraged to move in and provide assistance to customers with smaller volumes who do not wish to learn how to deal with the digital system of each container line. Not only are small customers unwilling to learn to use digital systems, but most frequently, each container line uses a different system. As a rule, forwarders are assumed to be quite competent in using all container lines’ digital platforms and can add this service to the product they sell to the cargo owner who does not wish to deal with the container line’ digitalisation.

#### The container bill of lading: Electronic or paper?

What transpired by interviews carried out with professionals from major container lines, a leading roro car carrier, and letter of credit specialists in banks is that the advent of the electronic bill of lading (e-bill of lading) will mainly improve processes where the bill of lading functions as a negotiable instrument transferring cargo ownership where banks are involved, and the cargo serves as collateral, namely a means of financing trade (Michael 2022). The obvious momentum currently in existence (Tan and Starr 2020) will most likely provide results, and the e-bill of lading will become more widely available and acceptable. The leading container lines have already implemented eBL applications supported by blockchain technology, TradeLens (tradelens.com) and WAVE (www.wavebl.com). These systems are slowly being implemented on major trade routes and will doubtless be spreading everywhere, even though Maersk and IBM announced their decision to withdraw the TradeLens offerings and discontinue the platform. The willing support of exporters and importers, who have been seen to resist similar advancements in the past, remains in doubt. What remains to be seen is whether the container lines will allow a choice in the matter.

## Conclusions and recommendations

This qualitative case study approach showed that although digitalisation offers important advantages in the shipping business, many barriers should be overcome.

All interviewees admitted that any investment in digitalisation and associated technologies deliver competitive advantages and cost benefits for shipowners and managers. They consider that digital technologies (like blockchain, IoT, artificial intelligence, machine learning etc.), in combination with modern connectivity methods, may provide prevention of system failures, reduction of maintenance costs, a decrease of off-hire claims, the accuracy of the information, compliance with maritime regulations, control of security and availability of data. Further adoption of IoT, AI and machine learning in the shipping industry could facilitate these benefits for all stakeholders. In other words, digitalisation may bring a real revolution to shipping and chartering business in the near future.

Furthermore, many advantages can be derived from using an e-bill of lading. Among these are an increase in the speed and a reduction in the cost of transactions together with the elimination of the problem of late arrival of documents. However, the research revealed a lack of confidence in using e-bills of lading because existing systems are not secure and the underlying legal framework is inadequate. Traditional bills of lading are acceptable because users are satisfied that they give merchants the protection they need.

The main barriers to digitalisation are the high implementation costs, the low quality of offshore internet connections, the lack of investment initiatives, the low level of modern digital technology diffusion through the supply chain, the risk prevention and the legal barriers to e-bill of lading. So, even though digitalisation has already had a positive impact, there are real issues to overcome. However, despite the existing barriers, there is no doubt that cross-industry data sharing through digital means will create a more efficient chartering business environment in the bulk and liner markets.

Therefore, further evolution of digitalisation and digital transformation in shipping business generally and chartering business specifically, is considered a necessity for profitability and sustainability. The digitalisation transformation of the shipping industry does not necessarily mean a sea of change for every company in every part of the industry at the same time. Different business models will be affected in different ways, although all players in all market segments are expected to be impacted by digitalisation at some point.

Consequently, further research should be taken regarding the digitalisation in various sectors of shipping business in order the ensued advantages and disadvantages and the possible problems and barriers to be overcome.

## Data Availability

This is a qualitative research conducted in March 2021, involving fifteen shipping companies. More specifically, the survey included seven cases of shipping companies operating in the bulk market and eight companies operating in the liner market. Intensive individual interviews took place with a small number of respondents to explore their perspectives on the particular subject of digitalisation. Based on research ethics and for confidentiality reasons, the anonymity of the interviewees was kept. The data that support the findings of this study are available from the authors but restrictions apply to the availability of these data, which were used under license for the current study, and so are not publicly available.

## Abbreviations

AI: Artificial Intelligence
E-BL or E-Bill of Lading: Electronic Bill of Lading
EDI: Electronic Data Interchange
ERP: Enterprise Resource Planning
IoT: Internet of Things
IT: Information Technology
ISO: International Organization for Standardization
SSAE: Statement on Standards for Attestation Engagements
